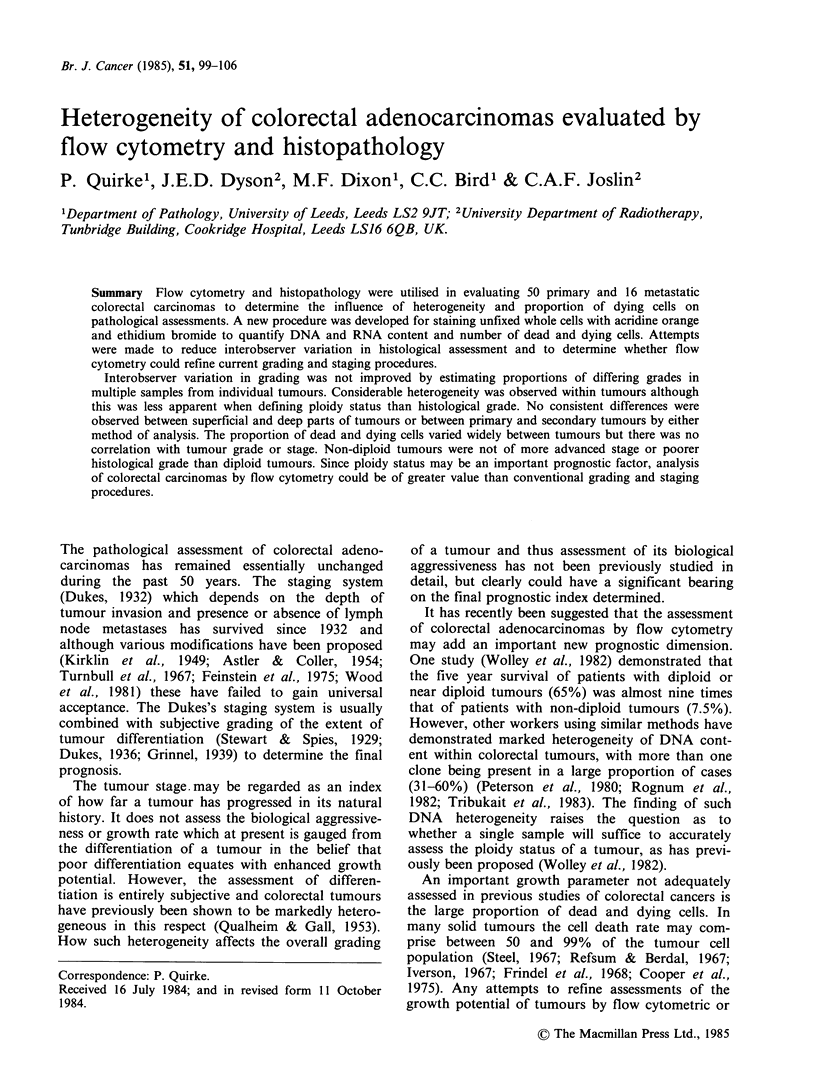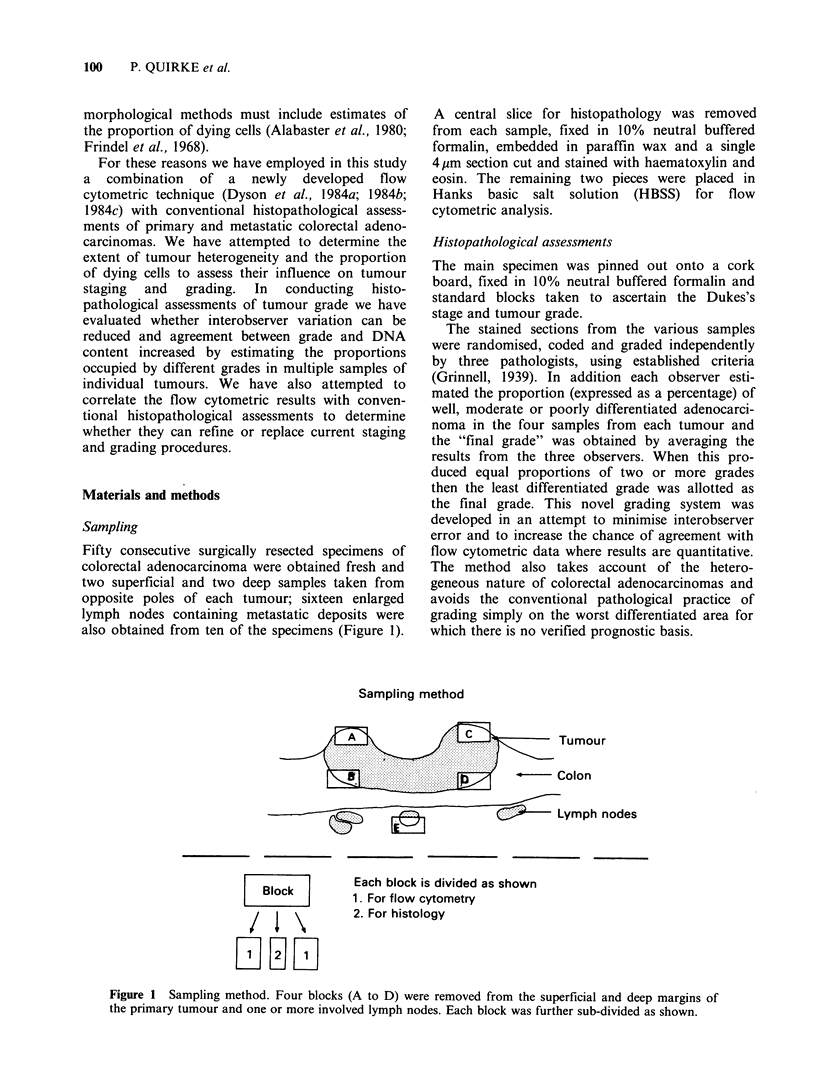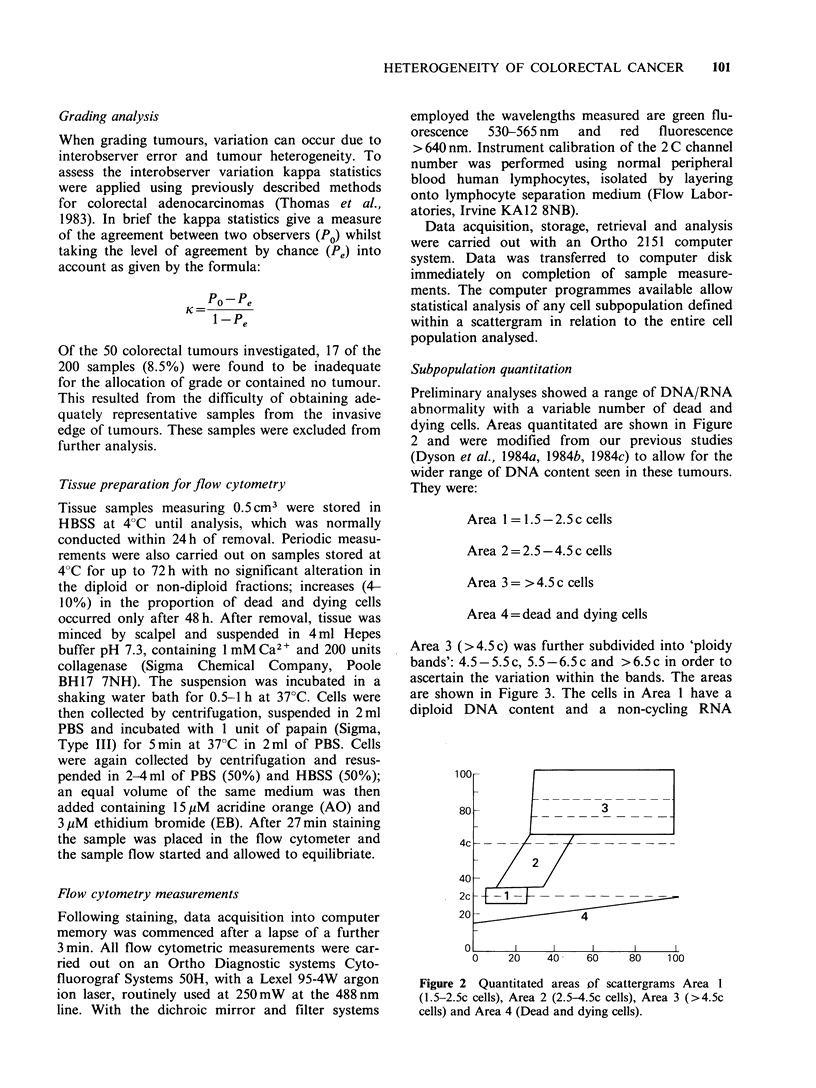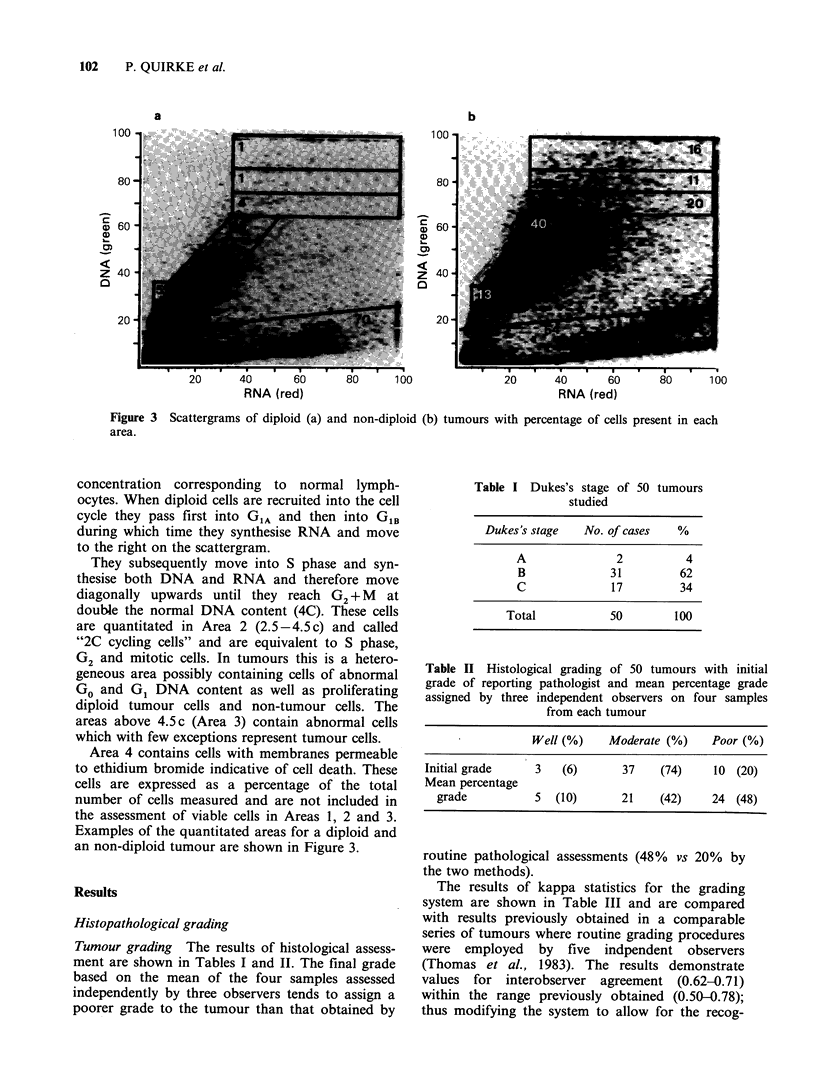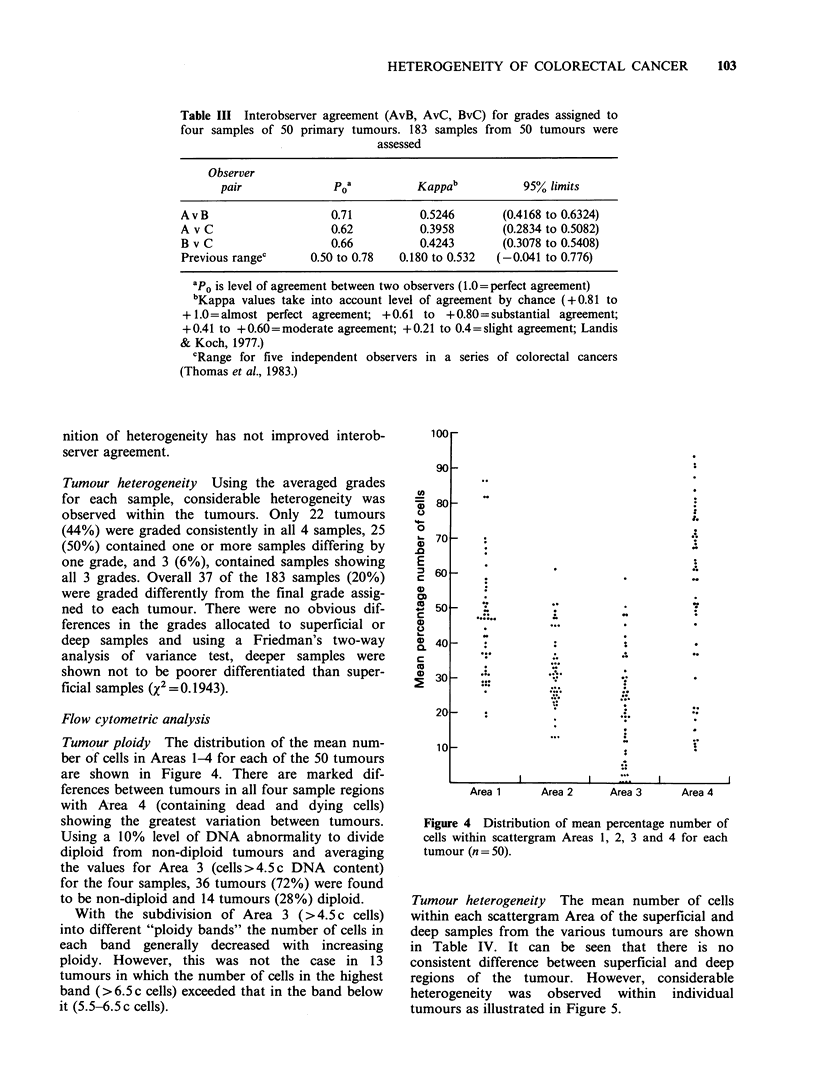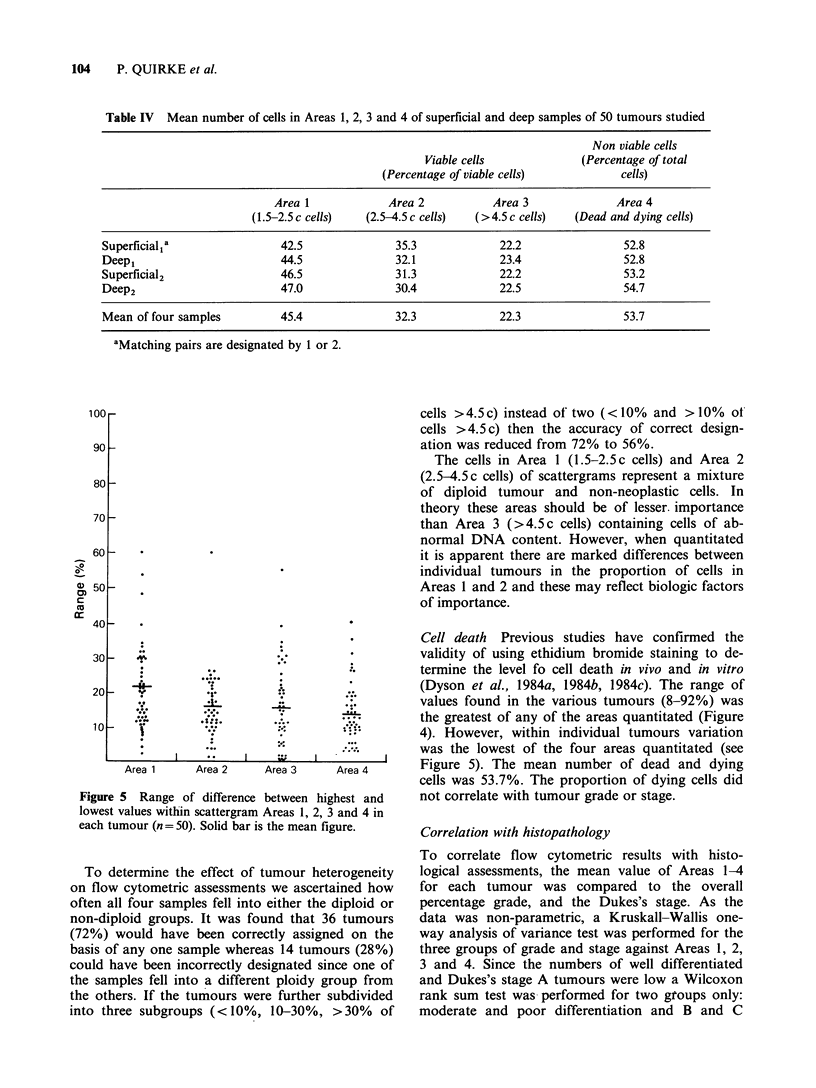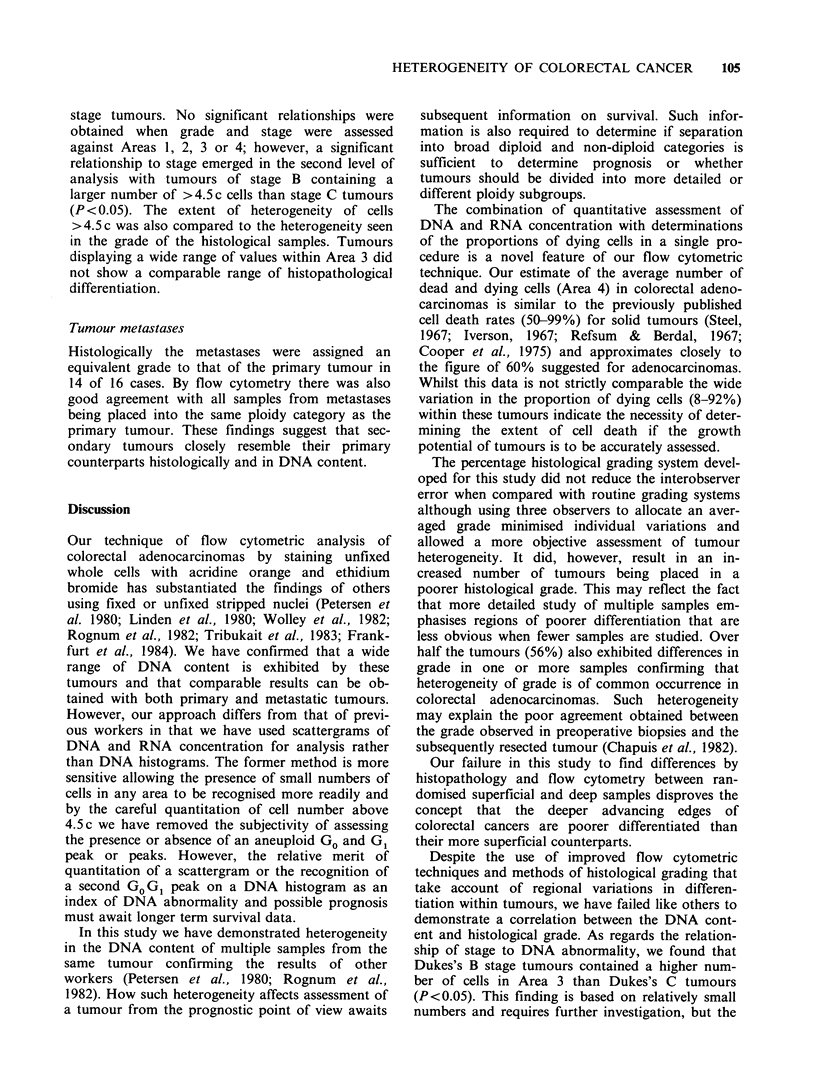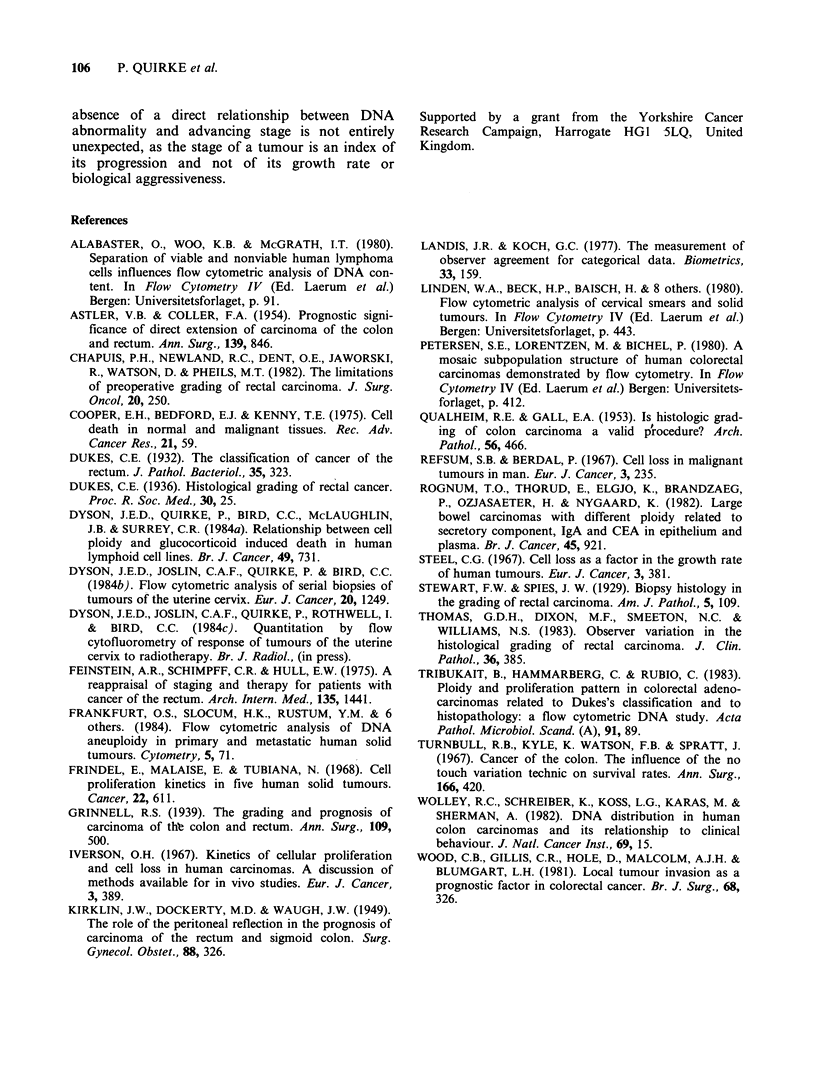# Heterogeneity of colorectal adenocarcinomas evaluated by flow cytometry and histopathology.

**DOI:** 10.1038/bjc.1985.14

**Published:** 1985-01

**Authors:** P. Quirke, J. E. Dyson, M. F. Dixon, C. C. Bird, C. A. Joslin

## Abstract

**Images:**


*Br. J. Cancer* (1985), **51**, 99–106

### Heterogeneity of colorectal adenocarcinomas evaluated by flow cytometry and histopathology

P. Quirke[1], J.E.D. Dyson[2], M.F. Dixon[1], C.C. Bird[1] & C.A.F. Joslin[2]

[1]*Department of Pathology, University of Leeds, Leeds LS2 9JT;* [2]*University Department of Radiotherapy, Tunbridge Building, Cookridge Hospital, Leeds LS16 6QB, UK.*

**Summary** Flow cytometry and histopathology were utilised in evaluating 50 primary and 16 metastatic colorectal carcinomas to determine the influence of heterogeneity and proportion of dying cells on pathological assessments. A new procedure was developed for staining unfixed whole cells with acridine orange and ethidium bromide to quantify DNA and RNA content and number of dead and dying cells. Attempts were made to reduce interobserver variation in histological assessment and to determine whether flow cytometry could refine current grading and staging procedures.

Interobserver variation in grading was not improved by estimating proportions of differing grades in multiple samples from individual tumours. Considerable heterogeneity was observed within tumours although this was less apparent when defining ploidy status than histological grade. No consistent differences were observed between superficial and deep parts of tumours or between primary and secondary tumours by either method of analysis. The proportion of dead and dying cells varied widely between tumours but there was no correlation with tumour grade or stage. Non-diploid tumours were not of more advanced stage or poorer histological grade than diploid tumours. Since ploidy status may be an important prognostic factor, analysis of colorectal carcinomas by flow cytometry could be of greater value than conventional grading and staging procedures.

The pathological assessment of colorectal adenocarcinomas has remained essentially unchanged during the past 50 years. The staging system (Dukes, 1932) which depends on the depth of tumour invasion and presence or absence of lymph node metastases has survived since 1932 and although various modifications have been proposed (Kirklin *et al.*, 1949; Astler & Coller, 1954; Turnbull *et al.*, 1967; Feinstein *et al.*, 1975; Wood *et al.*, 1981) these have failed to gain universal acceptance. The Dukes's staging system is usually combined with subjective grading of the extent of tumour differentiation (Stewart & Spies, 1929; Dukes, 1936; Grinnel, 1939) to determine the final prognosis.

The tumour stage may be regarded as an index of how far a tumour has progressed in its natural history. It does not assess the biological aggressiveness or growth rate which at present is gauged from the differentiation of a tumour in the belief that poor differentiation equates with enhanced growth potential. However, the assessment of differentiation is entirely subjective and colorectal tumours have previously been shown to be markedly heterogeneous in this respect (Qualheim & Gall, 1953). How such heterogeneity affects the overall grading of a tumour and thus assessment of its biological aggressiveness has not been previously studied in detail, but clearly could have a significant bearing on the final prognostic index determined.

Correspondence: P. Quirke.
Received 16 July 1984; and in revised form 11 October 1984.

It has recently been suggested that the assessment of colorectal adenocarcinomas by flow cytometry may add an important new prognostic dimension. One study (Wolley *et al.*, 1982) demonstrated that the five year survival of patients with diploid or near diploid tumours (65%) was almost nine times that of patients with non-diploid tumours (7.5%). However, other workers using similar methods have demonstrated marked heterogeneity of DNA content within colorectal tumours, with more than one clone being present in a large proportion of cases (31–60%) (Peterson *et al.*, 1980; Rognum *et al.*, 1982; Tribukait *et al.*, 1983). The finding of such DNA heterogeneity raises the question as to whether a single sample will suffice to accurately assess the ploidy status of a tumour, as has previously been proposed (Wolley *et al.*, 1982).

An important growth parameter not adequately assessed in previous studies of colorectal cancers is the large proportion of dead and dying cells. In many solid tumours the cell death rate may comprise between 50 and 99% of the tumour cell population (Steel, 1967; Refsum & Berdal, 1967; Iverson, 1967; Frindel *et al.*, 1968; Cooper *et al.*, 1975). Any attempts to refine assessments of the growth potential of tumours by flow cytometric or

© The Macmillan Press Ltd., 1985

morphological methods must include estimates of the proportion of dying cells (Alabaster *et al.*, 1980; Frindel *et al.*, 1968).

For these reasons we have employed in this study a combination of a newly developed flow cytometric technique (Dyson *et al.*, 1984*a*; 1984*b*; 1984*c*) with conventional histopathological assessments of primary and metastatic colorectal adenocarcinomas. We have attempted to determine the extent of tumour heterogeneity and the proportion of dying cells to assess their influence on tumour staging and grading. In conducting histopathological assessments of tumour grade we have evaluated whether interobserver variation can be reduced and agreement between grade and DNA content increased by estimating the proportions occupied by different grades in multiple samples of individual tumours. We have also attempted to correlate the flow cytometric results with conventional histopathological assessments to determine whether they can refine or replace current staging and grading procedures.

### Materials and methods

### *Sampling*

Fifty consecutive surgically resected specimens of colorectal adenocarcinoma were obtained fresh and two superficial and two deep samples taken from opposite poles of each tumour; sixteen enlarged lymph nodes containing metastatic deposits were also obtained from ten of the specimens (Figure 1). A central slice for histopathology was removed from each sample, fixed in 10% neutral buffered formalin, embedded in paraffin wax and a single 4 $\mu$m section cut and stained with haematoxylin and eosin. The remaining two pieces were placed in Hanks basic salt solution (HBSS) for flow cytometric analysis.

### *Histopathological assessments*

The main specimen was pinned out onto a cork board, fixed in 10% neutral buffered formalin and standard blocks taken to ascertain the Dukes's stage and tumour grade.

The stained sections from the various samples were randomised, coded and graded independently by three pathologists, using established criteria (Grinnell, 1939). In addition each observer estimated the proportion (expressed as a percentage) of well, moderate or poorly differentiated adenocarcinoma in the four samples from each tumour and the "final grade" was obtained by averaging the results from the three observers. When this produced equal proportions of two or more grades then the least differentiated grade was allotted as the final grade. This novel grading system was developed in an attempt to minimise interobserver error and to increase the chance of agreement with flow cytometric data where results are quantitative. The method also takes account of the heterogeneous nature of colorectal adenocarcinomas and avoids the conventional pathological practice of grading simply on the worst differentiated area for which there is no verified prognostic basis.

**Figure 1** Sampling method. Four blocks (A to D) were removed from the superficial and deep margins of the primary tumour and one or more involved lymph nodes. Each block was further sub-divided as shown.

*Grading analysis*

When grading tumours, variation can occur due to interobserver error and tumour heterogeneity. To assess the interobserver variation kappa statistics were applied using previously described methods for colorectal adenocarcinomas (Thomas *et al.*, 1983). In brief the kappa statistics give a measure of the agreement between two observers ($P_0$) whilst taking the level of agreement by chance ($P_e$) into account as given by the formula:

$$\kappa = \frac{P_0 - P_e}{1 - P_e}$$

Of the 50 colorectal tumours investigated, 17 of the 200 samples (8.5%) were found to be inadequate for the allocation of grade or contained no tumour. This resulted from the difficulty of obtaining adequately representative samples from the invasive edge of tumours. These samples were excluded from further analysis.

*Tissue preparation for flow cytometry*

Tissue samples measuring $0.5\,cm^3$ were stored in HBSS at 4°C until analysis, which was normally conducted within 24 h of removal. Periodic measurements were also carried out on samples stored at 4°C for up to 72 h with no significant alteration in the diploid or non-diploid fractions; increases (4–10%) in the proportion of dead and dying cells occurred only after 48 h. After removal, tissue was minced by scalpel and suspended in 4 ml Hepes buffer pH 7.3, containing 1 mM $Ca^{2+}$ and 200 units collagenase (Sigma Chemical Company, Poole BH17 7NH). The suspension was incubated in a shaking water bath for 0.5–1 h at 37°C. Cells were then collected by centrifugation, suspended in 2 ml PBS and incubated with 1 unit of papain (Sigma, Type III) for 5 min at 37°C in 2 ml of PBS. Cells were again collected by centrifugation and resuspended in 2–4 ml of PBS (50%) and HBSS (50%); an equal volume of the same medium was then added containing 15 μM acridine orange (AO) and 3 μM ethidium bromide (EB). After 27 min staining the sample was placed in the flow cytometer and the sample flow started and allowed to equilibriate.

*Flow cytometry measurements*

Following staining, data acquisition into computer memory was commenced after a lapse of a further 3 min. All flow cytometric measurements were carried out on an Ortho Diagnostic systems Cytofluorograf Systems 50H, with a Lexel 95-4W argon ion laser, routinely used at 250 mW at the 488 nm line. With the dichroic mirror and filter systems employed the wavelengths measured are green fluorescence 530–565 nm and red fluorescence >640 nm. Instrument calibration of the 2 C channel number was performed using normal peripheral blood human lymphocytes, isolated by layering onto lymphocyte separation medium (Flow Laboratories, Irvine KA12 8NB).

Data acquisition, storage, retrieval and analysis were carried out with an Ortho 2151 computer system. Data was transferred to computer disk immediately on completion of sample measurements. The computer programmes available allow statistical analysis of any cell subpopulation defined within a scattergram in relation to the entire cell population analysed.

*Subpopulation quantitation*

Preliminary analyses showed a range of DNA/RNA abnormality with a variable number of dead and dying cells. Areas quantitated are shown in Figure 2 and were modified from our previous studies (Dyson *et al.*, 1984*a*, 1984*b*, 1984*c*) to allow for the wider range of DNA content seen in these tumours. They were:

Area 1 = 1.5 − 2.5 c cells

Area 2 = 2.5 − 4.5 c cells

Area 3 = >4.5 c cells

Area 4 = dead and dying cells

Area 3 (>4.5 c) was further subdivided into 'ploidy bands': 4.5 − 5.5 c, 5.5 − 6.5 c and >6.5 c in order to ascertain the variation within the bands. The areas are shown in Figure 3. The cells in Area 1 have a diploid DNA content and a non-cycling RNA

**Figure 2** Quantitated areas of scattergrams Area 1 (1.5–2.5c cells), Area 2 (2.5–4.5c cells), Area 3 (>4.5c cells) and Area 4 (Dead and dying cells).

**Figure 3** Scattergrams of diploid (a) and non-diploid (b) tumours with percentage of cells present in each area.

concentration corresponding to normal lymphocytes. When diploid cells are recruited into the cell cycle they pass first into $G_{1A}$ and then into $G_{1B}$ during which time they synthesise RNA and move to the right on the scattergram.

They subsequently move into S phase and synthesise both DNA and RNA and therefore move diagonally upwards until they reach $G_2+M$ at double the normal DNA content (4C). These cells are quantitated in Area 2 (2.5−4.5c) and called "2C cycling cells" and are equivalent to S phase, $G_2$ and mitotic cells. In tumours this is a heterogeneous area possibly containing cells of abnormal $G_0$ and $G_1$ DNA content as well as proliferating diploid tumour cells and non-tumour cells. The areas above 4.5c (Area 3) contain abnormal cells which with few exceptions represent tumour cells.

Area 4 contains cells with membranes permeable to ethidium bromide indicative of cell death. These cells are expressed as a percentage of the total number of cells measured and are not included in the assessment of viable cells in Areas 1, 2 and 3. Examples of the quantitated areas for a diploid and an non-diploid tumour are shown in Figure 3.

### Results

### *Histopathological grading*

*Tumour grading* The results of histological assessment are shown in Tables I and II. The final grade based on the mean of the four samples assessed independently by three observers tends to assign a poorer grade to the tumour than that obtained by routine pathological assessments (48% *vs* 20% by the two methods).

The results of kappa statistics for the grading system are shown in Table III and are compared with results previously obtained in a comparable series of tumours where routine grading procedures were employed by five indpendent observers (Thomas *et al.*, 1983). The results demonstrate values for interobserver agreement (0.62–0.71) within the range previously obtained (0.50–0.78); thus modifying the system to allow for the recog-

**Table I** Dukes's stage of 50 tumours studied

| *Dukes's stage* | *No. of cases* | % |
|---|---|---|
| A | 2 | 4 |
| B | 31 | 62 |
| C | 17 | 34 |
| Total | 50 | 100 |

**Table II** Histological grading of 50 tumours with initial grade of reporting pathologist and mean percentage grade assigned by three independent observers on four samples from each tumour

| | *Well* (%) | | *Moderate* (%) | | *Poor* (%) | |
|---|---|---|---|---|---|---|
| Initial grade | 3 | (6) | 37 | (74) | 10 | (20) |
| Mean percentage grade | 5 | (10) | 21 | (42) | 24 | (48) |

**Table III** Interobserver agreement (AvB, AvC, BvC) for grades assigned to four samples of 50 primary tumours. 183 samples from 50 tumours were assessed

| *Observer pair* | $P_0$[a] | *Kappa*[b] | *95% limits* |
|---|---|---|---|
| A v B | 0.71 | 0.5246 | (0.4168 to 0.6324) |
| A v C | 0.62 | 0.3958 | (0.2834 to 0.5082) |
| B v C | 0.66 | 0.4243 | (0.3078 to 0.5408) |
| Previous range[c] | 0.50 to 0.78 | 0.180 to 0.532 | (−0.041 to 0.776) |

[a]$P_0$ is level of agreement between two observers (1.0 = perfect agreement)

[b]Kappa values take into account level of agreement by chance (+0.81 to +1.0 = almost perfect agreement; +0.61 to +0.80 = substantial agreement; +0.41 to +0.60 = moderate agreement; +0.21 to 0.4 = slight agreement; Landis & Koch, 1977.)

[c]Range for five independent observers in a series of colorectal cancers (Thomas *et al.*, 1983.)

nition of heterogeneity has not improved interobserver agreement.

*Tumour heterogeneity* Using the averaged grades for each sample, considerable heterogeneity was observed within the tumours. Only 22 tumours (44%) were graded consistently in all 4 samples, 25 (50%) contained one or more samples differing by one grade, and 3 (6%), contained samples showing all 3 grades. Overall 37 of the 183 samples (20%) were graded differently from the final grade assigned to each tumour. There were no obvious differences in the grades allocated to superficial or deep samples and using a Friedman's two-way analysis of variance test, deeper samples were shown not to be poorer differentiated than superficial samples ($\chi^2 = 0.1943$).

### *Flow cytometric analysis*

*Tumour ploidy* The distribution of the mean number of cells in Areas 1–4 for each of the 50 tumours are shown in Figure 4. There are marked differences between tumours in all four sample regions with Area 4 (containing dead and dying cells) showing the greatest variation between tumours. Using a 10% level of DNA abnormality to divide diploid from non-diploid tumours and averaging the values for Area 3 (cells > 4.5 c DNA content) for the four samples, 36 tumours (72%) were found to be non-diploid and 14 tumours (28%) diploid.

With the subdivision of Area 3 (> 4.5 c cells) into different "ploidy bands" the number of cells in each band generally decreased with increasing ploidy. However, this was not the case in 13 tumours in which the number of cells in the highest band (> 6.5 c cells) exceeded that in the band below it (5.5–6.5 c cells).

**Figure 4** Distribution of mean percentage number of cells within scattergram Areas 1, 2, 3 and 4 for each tumour ($n = 50$).

*Tumour heterogeneity* The mean number of cells within each scattergram Area of the superficial and deep samples from the various tumours are shown in Table IV. It can be seen that there is no consistent difference between superficial and deep regions of the tumour. However, considerable heterogeneity was observed within individual tumours as illustrated in Figure 5.

**Table IV** Mean number of cells in Areas 1, 2, 3 and 4 of superficial and deep samples of 50 tumours studied

| | | *Viable cells (Percentage of viable cells)* | | *Non viable cells (Percentage of total cells)* |
|---|---|---|---|---|
| | *Area* 1 (1.5–2.5 *c cells*) | *Area* 2 (2.5–4.5 *c cells*) | *Area* 3 (>4.5 *c cells*) | *Area* 4 (*Dead and dying cells*) |
| Superficial$_1$[a] | 42.5 | 35.3 | 22.2 | 52.8 |
| Deep$_1$ | 44.5 | 32.1 | 23.4 | 52.8 |
| Superficial$_2$ | 46.5 | 31.3 | 22.2 | 53.2 |
| Deep$_2$ | 47.0 | 30.4 | 22.5 | 54.7 |
| Mean of four samples | 45.4 | 32.3 | 22.3 | 53.7 |

[a]Matching pairs are designated by 1 or 2.

**Figure 5** Range of difference between highest and lowest values within scattergram Areas 1, 2, 3 and 4 in each tumour ($n=50$). Solid bar is the mean figure.

To determine the effect of tumour heterogeneity on flow cytometric assessments we ascertained how often all four samples fell into either the diploid or non-diploid groups. It was found that 36 tumours (72%) would have been correctly assigned on the basis of any one sample whereas 14 tumours (28%) could have been incorrectly designated since one of the samples fell into a different ploidy group from the others. If the tumours were further subdivided into three subgroups (<10%, 10–30%, >30% of cells >4.5c) instead of two (<10% and >10% of cells >4.5c) then the accuracy of correct designation was reduced from 72% to 56%.

The cells in Area 1 (1.5–2.5c cells) and Area 2 (2.5–4.5c cells) of scattergrams represent a mixture of diploid tumour and non-neoplastic cells. In theory these areas should be of lesser importance than Area 3 (>4.5c cells) containing cells of abnormal DNA content. However, when quantitated it is apparent there are marked differences between individual tumours in the proportion of cells in Areas 1 and 2 and these may reflect biologic factors of importance.

*Cell death* Previous studies have confirmed the validity of using ethidium bromide staining to determine the level fo cell death *in vivo* and *in vitro* (Dyson *et al.*, 1984*a*, 1984*b*, 1984*c*). The range of values found in the various tumours (8–92%) was the greatest of any of the areas quantitated (Figure 4). However, within individual tumours variation was the lowest of the four areas quantitated (see Figure 5). The mean number of dead and dying cells was 53.7%. The proportion of dying cells did not correlate with tumour grade or stage.

*Correlation with histopathology*

To correlate flow cytometric results with histological assessments, the mean value of Areas 1–4 for each tumour was compared to the overall percentage grade, and the Dukes's stage. As the data was non-parametric, a Kruskall–Wallis one-way analysis of variance test was performed for the three groups of grade and stage against Areas 1, 2, 3 and 4. Since the numbers of well differentiated and Dukes's stage A tumours were low a Wilcoxon rank sum test was performed for two groups only: moderate and poor differentiation and B and C

stage tumours. No significant relationships were obtained when grade and stage were assessed against Areas 1, 2, 3 or 4; however, a significant relationship to stage emerged in the second level of analysis with tumours of stage B containing a larger number of >4.5 c cells than stage C tumours ($P<0.05$). The extent of heterogeneity of cells >4.5 c was also compared to the heterogeneity seen in the grade of the histological samples. Tumours displaying a wide range of values within Area 3 did not show a comparable range of histopathological differentiation.

*Tumour metastases*

Histologically the metastases were assigned an equivalent grade to that of the primary tumour in 14 of 16 cases. By flow cytometry there was also good agreement with all samples from metastases being placed into the same ploidy category as the primary tumour. These findings suggest that secondary tumours closely resemble their primary counterparts histologically and in DNA content.

### Discussion

Our technique of flow cytometric analysis of colorectal adenocarcinomas by staining unfixed whole cells with acridine orange and ethidium bromide has substantiated the findings of others using fixed or unfixed stripped nuclei (Petersen *et al.* 1980; Linden *et al.*, 1980; Wolley *et al.*, 1982; Rognum *et al.*, 1982; Tribukait *et al.*, 1983; Frankfurt *et al.*, 1984). We have confirmed that a wide range of DNA content is exhibited by these tumours and that comparable results can be obtained with both primary and metastatic tumours. However, our approach differs from that of previous workers in that we have used scattergrams of DNA and RNA concentration for analysis rather than DNA histograms. The former method is more sensitive allowing the presence of small numbers of cells in any area to be recognised more readily and by the careful quantitation of cell number above 4.5 c we have removed the subjectivity of assessing the presence or absence of an aneuploid $G_0$ and $G_1$ peak or peaks. However, the relative merit of quantitation of a scattergram or the recognition of a second $G_0G_1$ peak on a DNA histogram as an index of DNA abnormality and possible prognosis must await longer term survival data.

In this study we have demonstrated heterogeneity in the DNA content of multiple samples from the same tumour confirming the results of other workers (Petersen *et al.*, 1980; Rognum *et al.*, 1982). How such heterogeneity affects assessment of a tumour from the prognostic point of view awaits subsequent information on survival. Such information is also required to determine if separation into broad diploid and non-diploid categories is sufficient to determine prognosis or whether tumours should be divided into more detailed or different ploidy subgroups.

The combination of quantitative assessment of DNA and RNA concentration with determinations of the proportions of dying cells in a single procedure is a novel feature of our flow cytometric technique. Our estimate of the average number of dead and dying cells (Area 4) in colorectal adenocarcinomas is similar to the previously published cell death rates (50–99%) for solid tumours (Steel, 1967; Iverson, 1967; Refsum & Berdal, 1967; Cooper *et al.*, 1975) and approximates closely to the figure of 60% suggested for adenocarcinomas. Whilst this data is not strictly comparable the wide variation in the proportion of dying cells (8–92%) within these tumours indicate the necessity of determining the extent of cell death if the growth potential of tumours is to be accurately assessed.

The percentage histological grading system developed for this study did not reduce the interobserver error when compared with routine grading systems although using three observers to allocate an averaged grade minimised individual variations and allowed a more objective assessment of tumour heterogeneity. It did, however, result in an increased number of tumours being placed in a poorer histological grade. This may reflect the fact that more detailed study of multiple samples emphasises regions of poorer differentiation that are less obvious when fewer samples are studied. Over half the tumours (56%) also exhibited differences in grade in one or more samples confirming that heterogeneity of grade is of common occurrence in colorectal adenocarcinomas. Such heterogeneity may explain the poor agreement obtained between the grade observed in preoperative biopsies and the subsequently resected tumour (Chapuis *et al.*, 1982).

Our failure in this study to find differences by histopathology and flow cytometry between randomised superficial and deep samples disproves the concept that the deeper advancing edges of colorectal cancers are poorer differentiated than their more superficial counterparts.

Despite the use of improved flow cytometric techniques and methods of histological grading that take account of regional variations in differentiation within tumours, we have failed like others to demonstrate a correlation between the DNA content and histological grade. As regards the relationship of stage to DNA abnormality, we found that Dukes's B stage tumours contained a higher number of cells in Area 3 than Dukes's C tumours ($P<0.05$). This finding is based on relatively small numbers and requires further investigation, but the absence of a direct relationship between DNA abnormality and advancing stage is not entirely unexpected, as the stage of a tumour is an index of its progression and not of its growth rate or biological aggressiveness.

Supported by a grant from the Yorkshire Cancer Research Campaign, Harrogate HG1 5LQ, United Kingdom.

### References

ALABASTER, O., WOO, K.B. & McGRATH, I.T. (1980). Separation of viable and nonviable human lymphoma cells influences flow cytometric analysis of DNA content. In *Flow Cytometry IV* (Ed. Laerum *et al.*) Bergen: Universitetsforlaget, p. 91.

ASTLER, V.B. & COLLER, F.A. (1954). Prognostic significance of direct extension of carcinoma of the colon and rectum. *Ann. Surg.*, **139**, 846.

CHAPUIS, P.H., NEWLAND, R.C., DENT, O.E., JAWORSKI, R., WATSON, D. & PHEILS, M.T. (1982). The limitations of preoperative grading of rectal carcinoma. *J. Surg. Oncol*, **20**, 250.

COOPER, E.H., BEDFORD, E.J. & KENNY, T.E. (1975). Cell death in normal and malignant tissues. *Rec. Adv. Cancer Res.*, **21**, 59.

DUKES, C.E. (1932). The classification of cancer of the rectum. *J. Pathol. Bacteriol.*, **35**, 323.

DUKES, C.E. (1936). Histological grading of rectal cancer. *Proc. R. Soc. Med.*, **30**, 25.

DYSON, J.E.D., QUIRKE, P., BIRD, C.C., McLAUGHLIN, J.B. & SURREY, C.R. (1984*a*). Relationship between cell ploidy and glucocorticoid induced death in human lymphoid cell lines. *Br. J. Cancer*, **49**, 731.

DYSON, J.E.D., JOSLIN, C.A.F., QUIRKE, P. & BIRD, C.C. (1984*b*). Flow cytometric analysis of serial biopsies of tumours of the uterine cervix. *Eur. J. Cancer*, **20**, 1249.

DYSON, J.E.D., JOSLIN, C.A.F., QUIRKE, P., ROTHWELL, I. & BIRD, C.C. (1984*c*). Quantitation by flow cytofluorometry of response of tumours of the uterine cervix to radiotherapy. *Br. J. Radiol.*, (in press).

FEINSTEIN, A.R., SCHIMPFF, C.R. & HULL, E.W. (1975). A reappraisal of staging and therapy for patients with cancer of the rectum. *Arch. Intern. Med.*, **135**, 1441.

FRANKFURT, O.S., SLOCUM, H.K., RUSTUM, Y.M. & 6 others. (1984). Flow cytometric analysis of DNA aneuploidy in primary and metastatic human solid tumours. *Cytometry*, **5**, 71.

FRINDEL, E., MALAISE, E. & TUBIANA, N. (1968). Cell proliferation kinetics in five human solid tumours. *Cancer*, **22**, 611.

GRINNELL, R.S. (1939). The grading and prognosis of carcinoma of the colon and rectum. *Ann. Surg.*, **109**, 500.

IVERSON, O.H. (1967). Kinetics of cellular proliferation and cell loss in human carcinomas. A discussion of methods available for in vivo studies. *Eur. J. Cancer*, **3**, 389.

KIRKLIN, J.W., DOCKERTY, M.D. & WAUGH, J.W. (1949). The role of the peritoneal reflection in the prognosis of carcinoma of the rectum and sigmoid colon. *Surg. Gynecol. Obstet.*, **88**, 326.

LANDIS, J.R. & KOCH, G.C. (1977). The measurement of observer agreement for categorical data. *Biometrics*, **33**, 159.

LINDEN, W.A., BECK, H.P., BAISCH, H. & 8 others. (1980). Flow cytometric analysis of cervical smears and solid tumours. In *Flow Cytometry* IV (Ed. Laerum *et al.*) Bergen: Universitetsforlaget, p. 443.

PETERSEN, S.E., LORENTZEN, M. & BICHEL, P. (1980). A mosaic subpopulation structure of human colorectal carcinomas demonstrated by flow cytometry. In *Flow Cytometry* IV (Ed. Laerum *et al.*) Bergen: Universitetsforlaget, p. 412.

QUALHEIM, R.E. & GALL, E.A. (1953). Is histologic grading of colon carcinoma a valid procedure? *Arch. Pathol.*, **56**, 466.

REFSUM, S.B. & BERDAL, P. (1967). Cell loss in malignant tumours in man. *Eur. J. Cancer*, **3**, 235.

ROGNUM, T.O., THORUD, E., ELGJO, K., BRANDZAEG, P., OZJASAETER, H. & NYGAARD, K. (1982). Large bowel carcinomas with different ploidy related to secretory component, IgA and CEA in epithelium and plasma. *Br. J. Cancer*, **45**, 921.

STEEL, C.G. (1967). Cell loss as a factor in the growth rate of human tumours. *Eur. J. Cancer*, **3**, 381.

STEWART, F.W. & SPIES, J. W. (1929). Biopsy histology in the grading of rectal carcinoma. *Am. J. Pathol.*, **5**, 109.

THOMAS, G.D.H., DIXON, M.F., SMEETON, N.C. & WILLIAMS, N.S. (1983). Observer variation in the histological grading of rectal carcinoma. *J. Clin. Pathol.*, **36**, 385.

TRIBUKAIT, B., HAMMARBERG, C. & RUBIO, C. (1983). Ploidy and proliferation pattern in colorectal adenocarcinomas related to Dukes's classification and to histopathology: a flow cytometric DNA study. *Acta Pathol. Microbiol. Scand.* (A), **91**, 89.

TURNBULL, R.B., KYLE, K. WATSON, F.B. & SPRATT, J. (1967). Cancer of the colon. The influence of the no touch variation technic on survival rates. *Ann. Surg.*, **166**, 420.

WOLLEY, R.C., SCHREIBER, K., KOSS, L.G., KARAS, M. & SHERMAN, A. (1982). DNA distribution in human colon carcinomas and its relationship to clinical behaviour. *J. Natl. Cancer Inst.*, **69**, 15.

WOOD, C.B., GILLIS, C.R., HOLE, D., MALCOLM, A.J.H. & BLUMGART, L.H. (1981). Local tumour invasion as a prognostic factor in colorectal cancer. *Br. J. Surg.*, **68**, 326.